# Role of USG and CT in the Evaluation of Abdominal Trauma

**DOI:** 10.7759/cureus.96943

**Published:** 2025-11-16

**Authors:** Amruth VC, Ravi Kumar Yeli, Harish N, Naveen Sheelavant

**Affiliations:** 1 Department of Radiology, Shridevi Institute of Medical Sciences and Research Hospital, Tumkur, IND; 2 Department of Radiology, Shri B. M. Patil Medical College Hospital and Research Centre, BLDE (Deemed to be University), Vijayapura, IND; 3 Department of Radiology, Al-Ameen Medical College, Vijayapura, IND

**Keywords:** abdominal trauma, computed tomography (ct), imaging technology, trauma-related death, ultrasonography (usg)

## Abstract

Objectives

The study aimed to evaluate the diagnostic accuracy and imaging patterns of abdominal injuries using USG and CT in patients with blunt abdominal trauma. Specifically, it assessed the ability of USG and CT to detect hemoperitoneum, intraperitoneal visceral injuries, and solid organ injuries. Imaging findings from USG and CT were analyzed and compared with intraoperative findings to facilitate early and accurate diagnosis, thereby aiding clinicians in timely management and minimizing morbidity and mortality.

Methodology

Patients admitted to Shri B. M. Patil Medical College Hospital and Research Centre with a history of abdominal trauma between November 2019 and May 2021 were included. A total of 64 patients who underwent both USG and CT examinations were evaluated. In cases requiring surgical intervention, imaging findings were compared with intraoperative observations.

Results

Among the 64 patients, all (100%) had positive USG and CT findings of abdominal trauma. Of these, 19 (29.7%) patients underwent surgery, while 45 (70.3%) were managed conservatively. There was a marked male predominance, with 60 (93.7%) males and four (6.3%) females, yielding a male-to-female ratio of 15:1. The most commonly injured organs were the spleen in 35 (54.7%) patients and the liver in 28 (43.7%) patients.

Conclusions

USG is a dynamic, real-time imaging modality that avoids ionizing radiation and is inexpensive, rapid, portable, and suitable for bedside use in hemodynamically unstable patients or those with multiple injuries. Minimal patient preparation is required, and the procedure is painless. Emergency USG can therefore be employed as a first-line diagnostic tool in suspected blunt abdominal trauma. CT, however, remains a highly sensitive modality for diagnosing and classifying abdominal injuries and provides valuable guidance for clinical management.

## Introduction

Abdominal injuries account for approximately 12-15% of all trauma-related deaths [1,2]. Road traffic accidents are the leading cause of trauma (around 80% of cases), while falls (often occupational), recreational accidents, and interpersonal violence contribute to the remainder.

Common abdominal injuries include lacerations of the liver, spleen, and kidneys, as well as urological trauma and bowel injury or infarction. The use of ultrasound and CT in the early management of abdominal trauma has become increasingly valuable [3,4]. CT demonstrates a diagnostic accuracy of about 97% in blunt abdominal trauma [5,6]. In addition, abdominal CT can detect coexisting extra-abdominal injuries, such as pneumothorax, pelvic fractures, and spinal fractures, which may be missed on clinical examination [7].

With multidetector CT, faster scanning rates and narrow collimation enhance contrast opacification in the mesenteric, retroperitoneal, and portal arteries and within parenchymal organs. This improves the detection of organ injury and sites of active arterial bleeding. Because trauma patients often cannot hold their breath, the rapid acquisition of multislice scans further reduces breathing artifacts [1,8].

Despite the well-established role of imaging in trauma care, variability persists in the diagnostic performance and practical application of USG and CT in different clinical settings, particularly in resource-limited environments. There remains a need to evaluate and compare the accuracy of these imaging modalities against intraoperative findings to determine their reliability in the rapid diagnosis and management of abdominal injuries. This study aimed to evaluate the diagnostic accuracy and imaging patterns of abdominal injuries using USG and CT in patients with blunt abdominal trauma. Specifically, it assessed the ability of USG and CT to detect hemoperitoneum, intraperitoneal visceral injuries, and solid organ injuries. It compared the imaging findings with intraoperative results to enhance early and precise clinical decision-making.

## Materials and methods

Study design and setting

This prospective cross-sectional study was conducted at Shri B. M. Patil Medical College Hospital and Research Centre in Vijayapura, India, over 18 months, from November 2019 to May 2021.

Study population

All patients admitted with a history of abdominal trauma during the study period were considered for evaluation. A total of 64 patients who underwent both USG and CT were included in the final analysis.

Sample size calculation

The minimum required sample size was determined using the formula:

$n = \frac{Z^{2} \, p \, (1 - p)}{d^{2}},$

where Z represents the standard normal variate at a 95% confidence level (1.96), p is the expected prevalence of intra-abdominal injury detection by CT in trauma patients (assumed as 85% based on previous studies), and d is the allowable error (10%). Based on this calculation, the required sample size was 62. Accordingly, 64 patients were recruited.

Sampling technique

Patients were selected using purposive sampling. Demographic, clinical, and imaging data were documented using a structured proforma.

Inclusion and exclusion criteria

All patients presenting with abdominal trauma, irrespective of age and sex, were eligible for inclusion. Patients who were pregnant, had psychiatric illness, or presented in hypovolemic or hemorrhagic shock were excluded. Additional exclusions included cases in which both USG and CT findings were negative, patients unable to undergo both investigations, and those discharged before completion of the required imaging.

Imaging protocol

USG of the abdomen and pelvis was performed using a PHILIPS HD11 XE ultrasound system (Philips Healthcare, Best, The Netherlands) following standard trauma protocols. CT was performed on a SIEMENS SOMATOM SCOPE 32-slice scanner (Siemens Healthineers, Erlangen, Germany). Scan parameters included 120 kVp, 100-300 mAs, and 5 mm slice thickness, with retro-reconstruction at 1.25 mm. Bolus tracking was employed, with the tracker placed in the descending aorta at the level of the diaphragmatic dome and a threshold of 150 Hounsfield units. Arterial phase images were obtained three seconds after the threshold was reached, and portovenous phase images were acquired 60 seconds after contrast injection.

In adults, 100 ml of nonionic iodinated contrast (350 mg/ml) was administered intravenously at 3-4 ml/s using a pressure injector. In children, 2 ml/kg of nonionic iodinated contrast (300 mg/ml) was administered at 1.5-2 ml/s. Arterial phase imaging extended from the dome of the diaphragm to the aortic bifurcation, while portovenous phase imaging covered the diaphragm dome to the inferior margin of the pubic symphysis. In suspected genitourinary trauma, delayed images were obtained at 20 minutes.

Correlation with surgical findings

Intraoperative findings were recorded for patients undergoing exploratory laparotomy and directly correlated with preoperative USG and CT results to determine diagnostic accuracy.

Ethical considerations

The study was initiated after approval from the Institutional Ethics Committee of Shri B. M. Patil Medical College Hospital and Research Centre. Prior to participation, written informed consent was obtained from all patients or their legal guardians.

Statistical analysis

Data were entered into Microsoft Excel (Microsoft Corporation, Redmond, WA, USA) and analyzed using IBM SPSS Statistics for Windows, Version 25.0 (Released 2017; IBM Corp., Armonk, NY, USA). Descriptive statistics were used to summarize patient characteristics and imaging findings, including means, standard deviations, frequencies, and percentages. Diagnostic performance of USG and CT was assessed by calculating sensitivity, specificity, positive predictive value, negative predictive value, and overall diagnostic accuracy, with surgical findings as the reference standard. The chi-square test was used to compare categorical variables, and a p-value of <0.05 was considered statistically significant.

## Results

The most affected age group was 21-30 years with 19 (30%), followed by 31-40 years with 16 (25%), 11-20 years with 10 (16%), 41-50 years with 10 (15%), more than 50 years with eight (12%), and 0-10 years with one (2%) (χ² = 18.69, df = 5, p = 0.0022). Males accounted for 58 (91%), while females accounted for six (9%) (χ² = 42.25, df = 1, p < 0.0001). Road traffic accidents were the leading cause of injury with 48 (75%), followed by falls from height with 13 (20%). Fall of a heavy object, sports injury, and stab/assault were less frequent, each contributing 1 (2%) (χ² = 129.44, df = 4, p < 0.0001) (Table 1).

**Table 1:** Demographic profile of patients with abdominal trauma (n = 64) ^*^ p < 0.05 indicates statistical significance.

Variable	Total number, N (%)	Chi-square value	p-Value
Age (in years)		18.69 (df = 5)	0.0022^*^
0-10	1 (2%)		
11-20	10 (16%)		
21-30	19 (30%)		
31-40	16 (25%)		
41-50	10 (15%)		
>50	8 (12%)		
Sex		42.25 (df = 1)	<0.0001^*^
Male	58 (91%)		
Female	6 (9%)		
Mode of injury		129.44 (df = 4)	<0.0001^*^
Road traffic accidents	48 (75%)		
Fall from height	13 (20%)		
Fall of a heavy object	1 (2%)		
Sports injury	1 (2%)		
Stab/assault	1 (2%)		

The spleen was the most commonly involved organ, with 35 (55%), followed by the liver with 28 (44%) and the kidneys with eight (13%). Less frequent organ involvement included the urinary bladder in one (1.5%), the adrenal gland in one (1.5%), and the rectum in one (1.5%) (Table 2).

**Table 2:** Organ involvement in abdominal trauma (n = 64)

Organ involved	N (%)
Spleen	35 (55%)
Liver	28 (44%)
Kidney	8 (13%)
Urinary bladder	1 (1.5%)
Adrenal gland	1 (1.5%)
Rectum	1 (1.5%)

In splenic trauma (n = 35), lacerations were most frequent in 32 (94%), followed by hematomas in 30 (88%) and pedicle injury in one (3%) (χ² = 28.67, df = 2, p < 0.0001). In liver trauma (n = 28), lacerations were observed in 26 (93%) and hematomas in 24 (86%), with no significant difference between injury types (χ² = 0.08, df = 1, p = 0.777). Among renal injuries (n = 8), lacerations occurred in four (50%), while contusions and hematomas were seen in two (25%) each (χ² = 1.00, df = 2, p = 0.607) (Table 3).

**Table 3:** Types of solid organ injuries ^* ^p < 0.05 indicates statistical significance.

Organ	Type of injury	N (%)	Chi-square test	p-Value
Spleen (n = 35)	Hematoma	30 (88%)	28.67 (df = 2)	<0.0001^*^
	Laceration	32 (94%)		
	Pedicle injury	1 (1%)		
Liver (n = 28)	Hematoma	24 (86%)	0.08 (df = 1)	0.777
	Laceration	26 (93%)		
Kidney (n = 8)	Contusion	2 (25%)	1.00 (df = 2)	0.607
	Hematoma	2 (25%)		
	Laceration	4 (50%)		

In the 19 operated cases, CT findings correlated with intraoperative findings in 17 (90%), while two (10%) showed discrepancies. Liver injuries were identified in 28 (100%) on CT/surgery, with two (7%) under-graded on USG (Grade III misclassified as Grade IV). Splenic injuries were identified in 35 (100%) on CT/surgery, with two (6%) under-graded on USG (Grade II misclassified as Grade III). All eight (100%) renal injuries were consistent across USG and CT/surgery. One rectal injury and one adrenal injury were missed on USG but detected on CT. The single urinary bladder injury was identified consistently on both USG and CT (Table 4).

**Table 4:** Correlation of imaging with intraoperative findings (operated cases, n = 19)

Organ involved	USG (N)	CT/surgery (N)	Remarks
Liver	26	28	Two cases undergraded (Grade III → IV)
Spleen	33	35	Two cases undergraded (Grade II → III)
Kidney	6	8	Consistent
Rectum	0	1	Missed on USG
Bladder	1	1	Consistent
Adrenal	0	1	Missed on USG

Among the 64 patients, 56 (87%) had single-organ involvement, while eight (13%) had multiple organs affected. A total of 45 (70%) had conservative management, whereas 19 (30%) required surgical intervention. Overall, 61 (95%) improved, while three (5%) expired (Table 5).

**Table 5:** Clinical management and outcomes (n = 64)

Variable	Category	N (%)
Organs involved	Single organ	56 (87%)
	Multiple organs	8 (13%)
Treatment	Conservative	45 (70%)
	Surgical	19 (30%)
Outcome	Improved	61 (95%)
	Expired	3 (5%)

**Figure 1:**
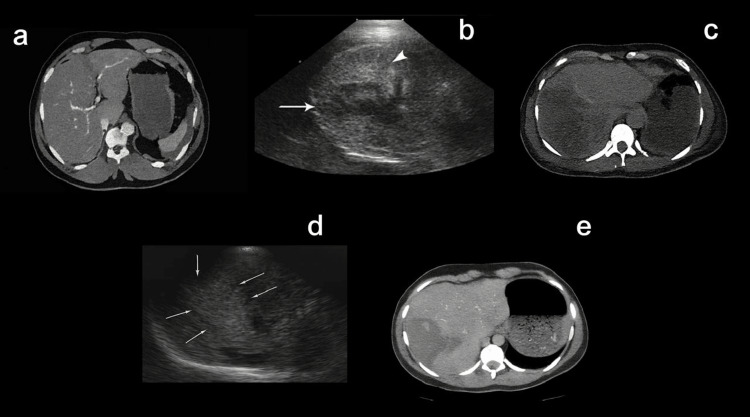
Imaging findings in liver trauma (a) Axial contrast-enhanced CT image showing a Grade II hepatic injury with a shallow parenchymal laceration and localized subcapsular hematoma. (b) USG image demonstrating a hypoechoic linear area (arrow) corresponding to a parenchymal laceration, with a focal hyperechoic area (arrowhead) suggestive of localized hematoma. (c) CT image showing a Grade III liver injury, characterized by a deeper laceration and associated perihepatic fluid collection. (d) Ultrasound image displaying multiple echogenic and hypoechoic areas (arrows) within the hepatic parenchyma, indicating heterogeneous echotexture and irregular margins consistent with parenchymal disruption and evolving hematoma formation. (e) Contrast-enhanced CT image revealing a large intraparenchymal hematoma with subcapsular extension, indicative of a Grade IV hepatic injury.

Liver injuries were common and varied in severity. Representative cases are shown in Figure 1, demonstrating the spectrum of hepatic trauma on USG and contrast-enhanced CT. Findings included Grade II injuries with hypodense parenchymal lacerations, Grade III injuries with more profound parenchymal disruption, and Grade IV injuries involving more than 25-50% of the hepatic lobe. Severe cases with Grade V injuries showed shattered hepatic parenchyma and associated hemoperitoneum. In addition, one patient presented with a Grade III liver injury accompanied by a Grade I right adrenal injury.

**Figure 2:**
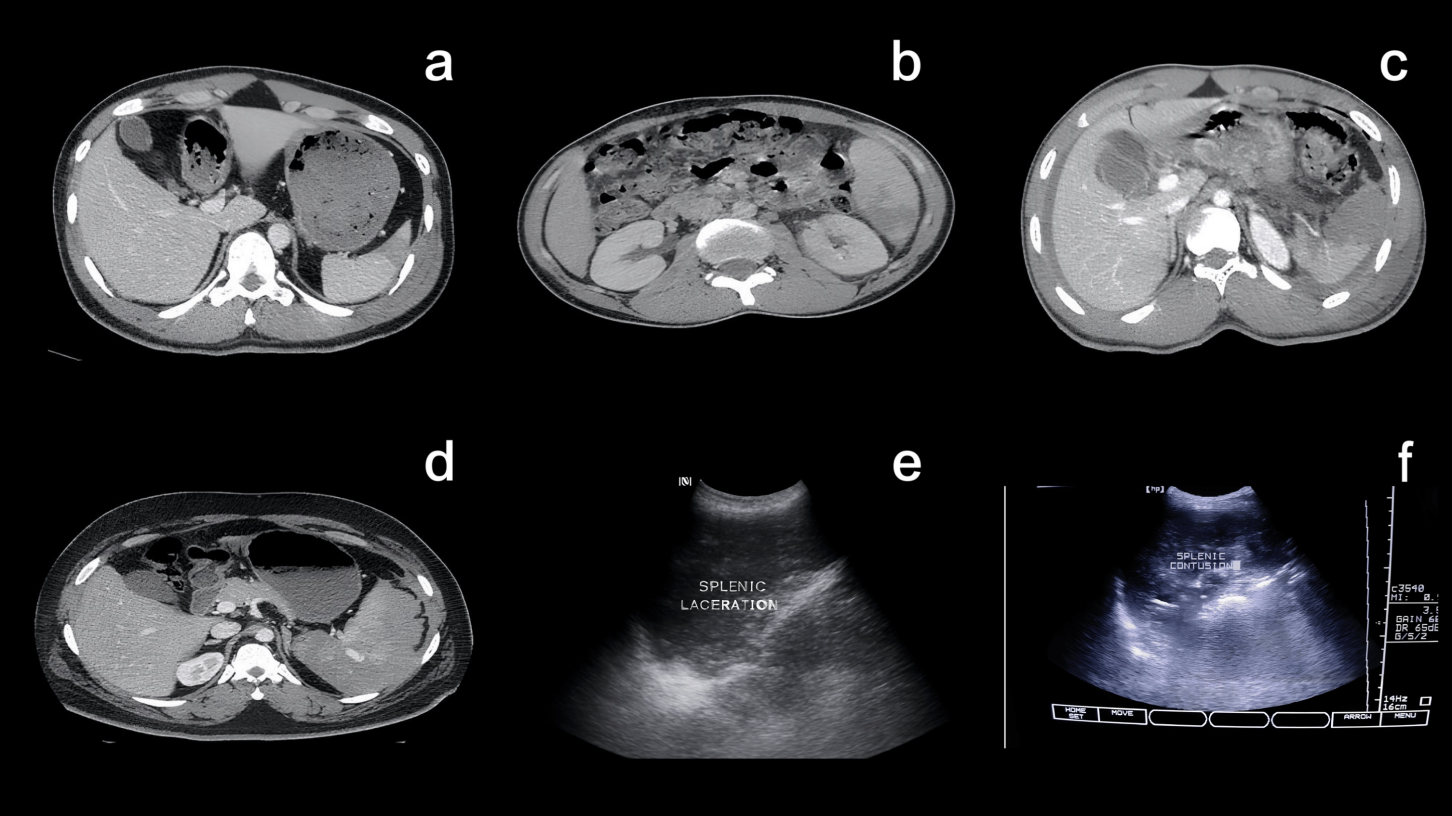
Imaging findings in splenic trauma (a) Contrast-enhanced CT axial image showing a Grade I splenic laceration with a linear hypodense defect in the parenchyma. (b) CT image demonstrating a Grade II splenic injury with deeper parenchymal disruption. (c) CT scan depicting a Grade III splenic laceration with associated perisplenic hematoma. (d) CT image of a Grade IV splenic injury showing extensive parenchymal disruption involving more than 25-50% of the splenic volume. (e) USG image showing a hypoechoic parenchymal defect consistent with splenic laceration. (f) USG image demonstrating diffuse hypoechoic changes suggestive of splenic contusion.

Splenic trauma was also frequently observed, with injuries ranging from minor to severe. Representative cases are shown in Figure 2, highlighting Grade I injuries with minor lacerations and hemoperitoneum, Grade II injuries with superficial parenchymal disruption, and Grade III injuries with deeper lacerations. More advanced cases included Grade IV injuries with complex parenchymal lacerations and Grade V injuries showing extensive splenic disruption with active contrast extravasation and ongoing bleeding, sometimes associated with splenic contusion.

**Figure 3:**
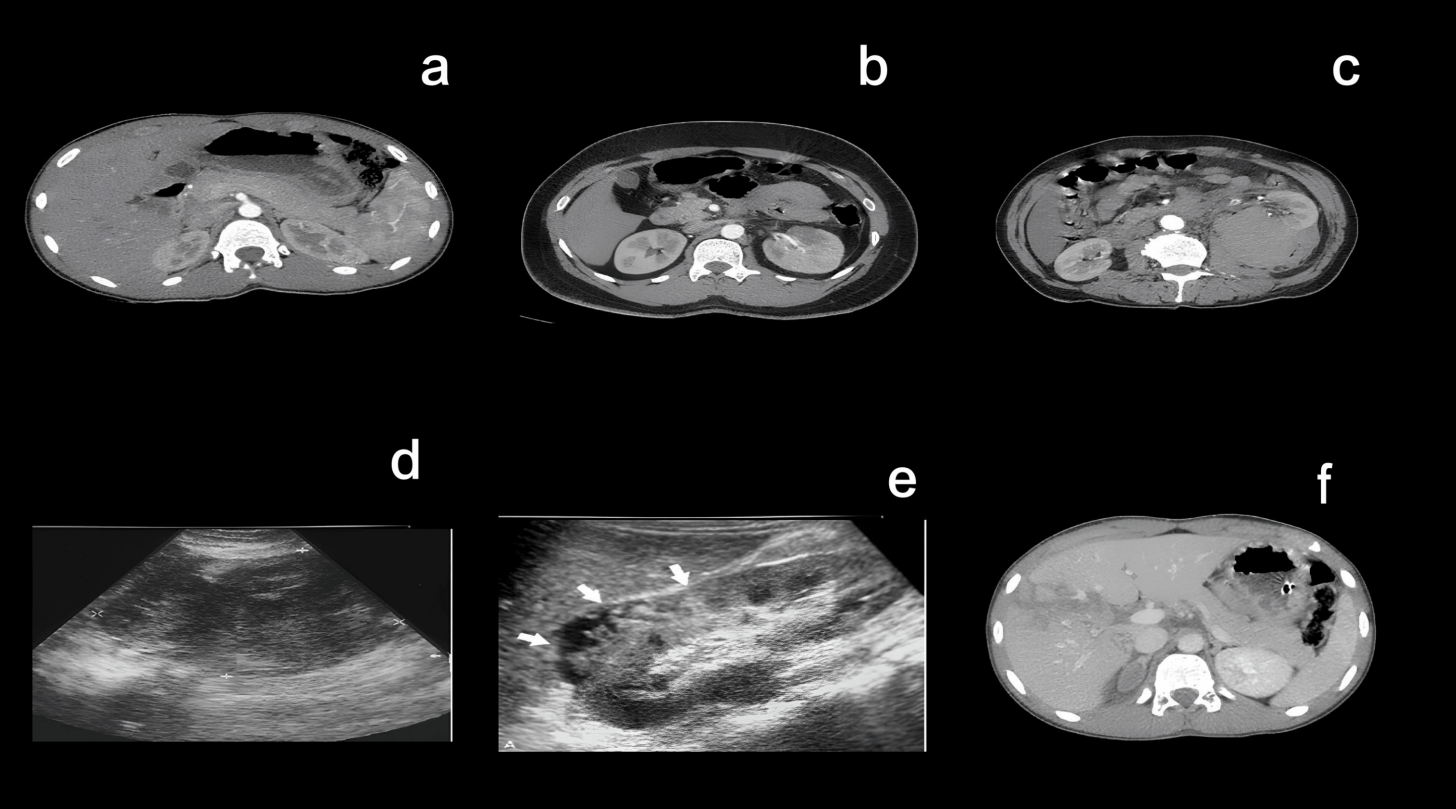
Imaging findings in renal trauma (a) Axial contrast-enhanced CT image showing a Grade I renal injury with a small subcapsular hematoma and intact renal parenchyma. (b) CT image demonstrating a Grade II renal injury characterized by a superficial cortical laceration (<1 cm depth) without urinary extravasation. (c) CT image showing a Grade III renal injury with a deeper laceration (>1 cm) extending into the medulla but without collecting system involvement. (d) USG image revealing a well-defined hypoechoic subcapsular collection, consistent with a small perinephric hematoma. (e) USG image displaying multiple hypoechoic and irregular areas (arrows) corresponding to parenchymal disruption and early liquefaction, suggestive of a higher-grade renal injury. (f) Contrast-enhanced CT image depicting a Grade IV renal injury with a deep laceration extending into the collecting system and perinephric fluid collection.

Renal injuries were less common but clinically significant. As demonstrated in Figure 3, these ranged from Grade I subcapsular hematomas to Grade II cortical lacerations confined to the parenchyma. Grade III renal injuries showed deeper lacerations, hemoperitoneum, and large perirenal hematomas. Severe cases included Grade IV injuries with extension into the collecting system, occasionally associated with pelvic free fluid and bladder wall defects indicating concurrent bladder injury.

**Figure 4:**
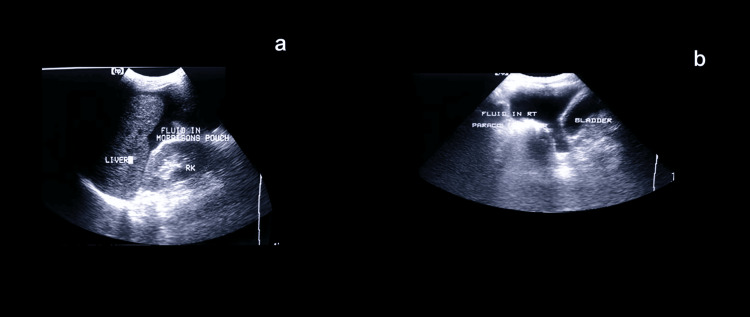
USG demonstrating hemoperitoneum (a) FAST image showing free fluid in Morrison’s pouch (hepatorenal recess) adjacent to the liver and right kidney. (b) Ultrasound image depicting free fluid in the right paracolic gutter surrounding the urinary bladder. FAST, Focused Assessment with Sonography for Trauma

Other uncommon injuries were also identified in isolated cases. One patient sustained a rectal injury with contrast extravasation into the peritoneal cavity, consistent with a Grade II rectal tear. Free fluid was also detected in Morrison’s pouch and the paracolic gutters on USG, as shown in Figure 4, underscoring the role of Focused Assessment with Sonography for Trauma (FAST) in rapidly detecting hemoperitoneum in trauma patients. Overall, CT proved superior in delineating the grade and extent of solid organ injuries, hemoperitoneum, and associated visceral damage. At the same time, USG remained valuable as a rapid bedside tool, particularly in unstable patients.

## Discussion

Abdominal trauma is a significant cause of morbidity and mortality worldwide, particularly in young adults, and timely diagnosis is essential for improving outcomes [9,10]. Imaging plays a pivotal role in evaluating such patients, with USG commonly employed as a rapid and noninvasive first-line tool, particularly in the form of FAST. However, its limitations in detecting and grading solid organ and hollow viscus injuries are well recognized [11]. CT, by contrast, is widely regarded as the gold standard for assessing abdominal trauma because of its superior sensitivity and specificity in organ injury characterization, hemoperitoneum detection, and injury grading [9,12]. The present study contributes to this evidence base by analyzing the role of USG and CT in abdominal trauma and comparing our findings with those of the previously reported literature [13,14].

In this study, the spleen and liver were the most frequently injured abdominal organs, accounting for 35 (55%) and 28 (44%) cases, respectively, followed by the kidneys in eight (13%), and the urinary bladder, rectum, and adrenal gland in one (1%) each. Hemoperitoneum was observed in all patients (100%). Among the 19 patients who underwent surgery, CT grading did not correspond with intraoperative grading in three (16%), while the remaining 45 (70%) patients were managed conservatively. The abnormalities detected on USG and CT included liver hematomas and lacerations; splenic hematomas, lacerations, pedicle injuries, and shattered spleen; renal contusions, hematomas, and lacerations; urinary bladder tears; adrenal hematomas; and rectal lacerations. These observations are consistent with previously reported patterns of abdominal trauma [13,14].

Most patients were young adults, with the highest incidence in the 21-30 age group (30%), followed by the 31-40 years age group (25%). Federle et al. [15] also reported a similar age distribution, with the highest incidence in the 21-30 years group (35%). While they described a male-to-female ratio of 13:7, our series showed a much higher male predominance (94:6), indicating a greater susceptibility of males to abdominal trauma in our setting. Regarding etiology, road traffic accidents were the leading cause in 48 (75%) patients, followed by falls from height in 13 (20%). Stab injuries, sports trauma, and heavy object injuries were uncommon, each accounting for one (2%). By contrast, Siddique et al. [16] reported stab wounds as the leading cause of abdominal trauma, followed by road traffic accidents, a difference likely explained by the location of our hospital near a national highway, resulting in higher referrals for RTA-related injuries.

Liver trauma in this series demonstrated excellent correlation between CT and surgical findings, with CT showing a sensitivity of 93% and no false positives. Four patients with grade IV liver injuries underwent surgery, whereas those with grade III or lower injuries were managed conservatively. One patient with a grade III liver trauma and a concomitant grade II rectal injury required surgery for the rectal lesion, while the liver injury was managed nonoperatively. Sonography underestimated severity in some cases: lacerations initially diagnosed as grade III were later confirmed as grade IV on CT and intraoperative evaluation. One laceration was missed entirely on ultrasound due to subcutaneous emphysema obscuring visualization. These findings support the work of Ravindernath and Reddy [12], who demonstrated the superiority of CT over USG in detecting and grading hepatic trauma.

Splenic trauma also showed a strong correlation between CT and intraoperative findings, although in two cases, injuries were underestimated because closely apposed lacerations were masked by large perilesional hematomas. Ultrasound underestimated two injuries initially graded as II but later confirmed as grade III on CT, and two splenic injuries were missed altogether, both in patients with concomitant grade IV liver trauma. In this study, ultrasound had a sensitivity of 94% for splenic injuries, with no false positives. Coccolini et al. [9] similarly emphasized CT as the gold standard for splenic trauma, with sensitivity and specificity approaching 100%. In contrast, USG is more likely to underestimate or miss injuries near the diaphragm.

Renal trauma was identified in eight patients, all managed conservatively. CT grading correlated well with surgical outcomes, but USG underestimated severity in two patients and missed two minor renal lacerations that were later confirmed intraoperatively. These findings highlight the limitations of ultrasound in renal trauma evaluation and align with the observations of Osman et al. [11], who stressed the reliability of CT in renal injury grading and management. Laal et al. [17], in their analysis of over 16,000 trauma patients, also reported that the majority of renal injuries were low grade, a trend similarly observed in our series.

Adrenal trauma was documented in one patient with associated hepatic and renal injuries. While CT clearly demonstrated the lesion, ultrasound failed to detect it because of bowel gas interference. The injury was managed conservatively without adverse outcomes. This is consistent with the findings of Badawy et al. [18], who noted both the rarity of adrenal trauma and the superiority of CT for its detection.

Rectal injury was encountered in one patient with concurrent splenic trauma. CT underestimated the lesion as grade II, whereas surgical exploration revealed a grade III circumferential tear. This underscores the limitations of CT in detecting hollow viscus and rectal injuries, a diagnostic challenge also discussed by Clark and Maine [19], who highlighted that clinical suspicion should remain high even with negative imaging.

Urinary bladder rupture was first suspected on ultrasound when the bladder failed to distend with saline infusion, and real-time imaging revealed leakage into the peritoneal cavity. CT subsequently confirmed the diagnosis by demonstrating a wall defect with extravasation of contrast and adjacent urine collection. These findings are consistent with the recommendations of Morey et al. [20], who advocated CT cystography as the gold standard for diagnosing bladder rupture.

Anderson et al. [21] reported that most abdominal injuries were of moderate grade, with grade II injuries being most common (45%), followed by grade III (21%) and grade IV (19%). In the present series, grade II injuries (38%) and grade III injuries (34%) were also most frequent, suggesting a predominance of moderate trauma, likely due to the high number of road traffic and fall-related injuries. Overall, the findings reaffirm that CT remains the gold standard for diagnosis and grading of abdominal trauma across solid organs, with superior sensitivity and specificity compared to USG. While USG is a valuable initial tool because of its accessibility, rapidity, and ability to detect free fluid, its limitations in grading and detecting specific injuries highlight the necessity of CT for definitive evaluation and management planning.

Limitations

This study has certain limitations that should be acknowledged. First, it was conducted at a single tertiary care center with a relatively small sample size of 64 patients, which may limit the generalizability of the findings. Using purposive sampling could have introduced selection bias, as patients unable to undergo both USG and CT were excluded, potentially overlooking critical presentations. Another limitation is that only 19 patients underwent surgical intervention, restricting the extent of intraoperative correlation and limiting the ability to validate imaging findings across the entire cohort. Furthermore, USG is highly operator dependent, and its diagnostic accuracy can be compromised by patient-related factors such as obesity, subcutaneous emphysema, or overlying bowel gas, which may result in underestimation or missed injuries. Although CT served as the reference standard, its reliance on intravenous contrast and ionizing radiation restricts repeated use, particularly in pediatric and pregnant patients excluded from this study. In addition, patients presenting in hypovolemic or hemorrhagic shock were not included, thereby underrepresenting the diagnostic challenges in unstable trauma cases. Finally, the study focused primarily on immediate diagnosis and short-term outcomes, without incorporating long-term follow-up to assess delayed complications or outcomes related to abdominal trauma.

## Conclusions

Road traffic accidents were the leading cause of blunt abdominal trauma. Males were predominantly affected, and cases were distributed across a wide age range. The spleen and liver were the most frequently injured organs, with hemoperitoneum observed in most patients. USG demonstrated good sensitivity for detecting solid organ injuries. In contrast, CT showed excellent correlation with intraoperative findings, reinforcing its role as a reliable preoperative tool for the grading and management of abdominal trauma.
